# The Royal Army Medical Corps: A Review of the Prospects and Pay

**Published:** 1914-09-05

**Authors:** 


### THE ROYAL ARMY MEDICAL CORPS.

### A Review of the Prospects and Pay.

Owing to the changes that recent years have wrought in its status and in its terms of pay and service, the Royal Army Medical Corps (R.A.M.C.) offers many attractions. Especially is this the case if a man joins immediately after taking his qualification. Admission to the Army Medical Corps is obtained by a competitive entrance examination, of which there are two annually, usually in January and July, the exact dates being announced each year by advertisement in the newspapers. To be eligible to compete in this examination a candidate must be between the ages of 21 and 28, and he must be registered under the Medical Act, so as to be qualified to practise medicine and surgery in Great Britain and Ireland.

### The First Thing to Do.

To obtain permission to take part in the examination a certain procedure has to be followed and certain conditions have to be fulfilled. The first thing the candidate has to do is to make application on a special form, to be obtained from the Secretary, War Office, Whitehall, London, S.W.

Having signed the declaration on the form as to his nationality, and that he is free from mental or bodily infirmity and has good vision, the candidate sends his application to the Director-General of the Army Medical Service, who then obtains from the dean, or other responsible authority, of the Medical School in which the candidate completed his course as a medical student a confidential report as to his character, conduct, professional ability, and fitness to hold a commission in the Royal Army Medical Corps. After the receipt of this report and after a personal interview with the candidate the Director-General decides as to whether he should be allowed to compete for a commission or not. If the decision is favourable, then permission is given to do so, provided he passes satisfactorily the physical examination. This physical examination is not a severe one, but is in every way a thorough and searching one. In it attention is directed to the correlation of age, height and chest measurement, and to the weight. A good deal of importance, too, is attached to the power of vision. The Army Test Types and Snellen's "Optotypi" (Edition 1902) are used for determining the visual acuteness. If a candidate can read D6 at 6 mètres (20 English feet) and D0.6 at any distance selected by himself, with each eye without glasses, he will be considered "Fit." At the same time a moderate degree of myopia is not considered a disqualification, provided that it can be corrected by glasses so as to secure adequate vision for the performance of operations, and that no organic disease exists. The other matters brought under observation in this physical examination are the hearing, absence of stuttering, the condition of the teeth (loss or decay of the teeth being considered disqualification), the state of the chest organs, the existence of rupture, of varicocele, of any chronic skin disease or any congenital malformation or defect, and of any evidence of previous acute or chronic disease. If the inquiries on all these points are satisfactory, the candidate is eligible to present himself at the next entrance examination on payment of a fee of £1.

### High Marks in the Examination.

This entrance examination is held in London, occupies about four days, and is conducted by an Examining Board, consisting of eight physicians and surgeons appointed from the civil hospitals and medical schools of the United Kingdom. The examination is in medicine and surgery, is of a clinical and practical character, and is partly written and partly oral. Thus, the candidate has to draw up a clinical report and also to furnish a commentary on a medical and surgical case submitted to him, and he has also to diagnose other clinical cases brought forward in the oral examination, which in medicine includes questions in pathology, and in surgery, diseases of the eye, with operative surgery and bandaging. The total number of marks obtained by each candidate in all the above subjects determines his place in the order of merit, and the number of appointments announced for competition is filled up from the list so arranged. In the case of candidates who have served in the Officers' Training Corps and are in possession of the certificates A and B a very handsome addition is made of what are known as service marks, certificate A carrying with it 100 marks additional, and A and B 200 marks. Such a decided increase may materially alter a candidate's position in the list of successful candidates, so that service in the Officers' Training Corps carries with it very material advantages.

A candidate who has been successful in the entrance examination at once gains admission to the Royal Army Medical Corps, and is appointed a lieutenant on probation, receiving during that time the pay of that rank. His commission has, however, to be confirmed, which is done if he successfully passes certain qualifying examinations held after he has gone through a definite course of instruction in recruiting duties, in military surgery, in tropical medicine, in hygiene, in pathology, in medical administration, in regimental duties, and in drill and field training. To allow of this being satisfactorily carried out he is required to attend, immediately on his appointment as probationary lieutenant, at the Royal Army Medical College, Millbank, London, and at the Royal Army Medical Corps School of Instruction at Aldershot. A concession has, however, been made by the military authorities to any candidate who at the time of passing the entrance examination holds, or is about to hold, a resident appointment in a recognised civil hospital. Should the candidate desire to fulfil the engagement he can be seconded for a period of a year to allow him to hold the appointment, and it has been decided that his future prospects in the Service will not suffer, except in one respect—namely, that he will receive no pay from Army funds during the time he holds his civil appointment.

During the probationary period of instruction held at the Royal Army Medical College and at Aldershot qualifying examinations are held at the end of each course in the different subjects, and those lieutenants who have gained the required marks, and who have satisfied the Director-General that they possess the necessary skill, knowledge, and character for permanent appointments, will have their commissions confirmed. Their precedence amongst each other will be in order of merit, and this will be settled by the combined results of the entrance examination and the examinations undergone while on probation, so that a candidate has the opportunity of improving his position while on probation. The result is that in this way the outcome of the probationary period of study may have some influence on a candidate's future from a service point of view. In connection with this order of precedence it may be mentioned here that a special rule applies to the lieutenants seconded to allow of their holding a resident medical appointment in a civil hospital. The place of a lieutenant so seconded will be determined by the place he has gained at the entrance examination. This he will retain. All that is required of him is that at the end of his hospital appointment he must attend the probationary courses of instruction at the Royal Army Medical College and at Aldershot and pass on their completion the qualifying examinations for the same. The public confirmation of every commission is announced in the *London Gazette*, and shortly after that each officer will find himself posted for duty in one of the military hospitals at home. In connection with this it is only fair to state that every newly appointed officer is given an opportunity of naming the command in which he would prefer to serve. What are the prospects opened up by this career?

### The Meaning of "Pay."

When speaking of the pay of an Army medical officer, it must be remembered that in addition to the actual daily money he receives he is provided by Government with a servant, with lodgings, and with fuel and light, so that all these latter must be taken into consideration in reckoning his annual emolument. Thus, the actual monetary pay of a lieutenant on joining is £255 10s., but with the allowances mentioned above it amounts to £326 annually, it being, however, understood that if Government provides accommodation in barracks and fuel and light the allowances for these are not given, so that under these circumstances the lieutenant will only get his £255 10s. plus an allowance of £18 5s. for a servant, which gives him £273 15s. per annum. Bearing this explanation in mind the following are the rates of pay and allowances for Army medical officers when serving at home, a different scale coming into force, as we shall see, when they do duty abroad.

| | Pay £ | s. | d. | With allowances £ | s. | d. |
|---|---|---|---|---|---|---|
| Lieutenant ... ... | 255 | 10 | 0 | 326 | 0 | 0 |
| Captain ... ... ... | 282 | 17 | 6 | 372 | 1 | 9 |
| Captain, after 7 years' full-pay service ... | 310 | 5 | 0 | 399 | 9 | 3 |
| Captain, after 10 years' full-pay service ... | 383 | 5 | 0 | 472 | 9 | 3 |
| Major ... ... ... | 428 | 17 | 6 | 583 | 9 | 7 |
| Major, after 3 years' service as such ... | 474 | 10 | 0 | 629 | 2 | 1 |
| Lieut.-Colonel ... ... | 547 | 10 | 0 | 711 | 4 | 7 |
| Lieut.-Colonel, after 3 years' service as such | 638 | 15 | 0 | 802 | 9 | 7 |
| Colonel ... ... ... | 821 | 5 | 0 | 1,008 | 16 | 1 |
| Surgeon-General ... | 1,095 | 0 | 0 | 1,447 | 16 | 3 |

Under certain conditions additional pay is given, as when a medical officer is in charge of a general or other hospital, and the higher staff appointments in the R.A.M.C. have their own special remuneration; the Director-General, for instance, receiving £2,000 per annum, while officers appointed professors and assistant-professors at the Royal Army Medical College receive the pay and allowances of their rank, plus £200 and £80 per annum respectively.

### Service Abroad.

The Army medical officer is, however, liable to be sent abroad, the number required for this foreign service varying from time to time. As a rule a lieutenant is usually sent abroad during the second year of his service, the greater majority of them going to India. The term of service abroad is five years for India, the Mediterranean, and South Africa, and three years at other stations. When an officer's turn arrives for foreign service he is duly warned some months before he is required to embark, and has the opportunity of naming his preference for any particular station abroad, his wishes being met as far as Service exigencies permit. When stationed abroad an Army medical officer receives a higher scale of pay. Thus, in India a lieutenant receives 420 rupees per month, which represents annual pay of about £500 if the rupee is reckoned at its supposed value of two shillings. Other ranks show a corresponding increase, and in view of the fact that service in India in the present day is passed under much more favourable climatic and hygienic conditions than formerly prevailed, by many it is preferred to service at home and has many advantages. This is seen in the very keen competition which prevails for what is known as "The Indian Medical Service," which is quite distinct from the R.A.M.C., the members of it undertaking to serve continuously in India with what is called "The Indian Army," made up of both British and native troops. The qualifying conditions and examinations (compare the article on page 626) are very much the same as for the R.A.M.C., but the officers on appointment are at once sent to India by troopship or otherwise at the public cost and they spend all their service abroad. Naturally the pay and allowances, the leave and rules, the invalid and retiring pensions, and the half-pay are different from those of the home service, and are, speaking generally, on a higher scale.

Full particulars can be obtained by application to the Military Secretary, India Office, London, S.W.

Another fact that should be mentioned is that officers of the R.A.M.C. are permitted to accept employment under the Foreign or Colonial Offices when the interests of the public service make it desirable. By this arrangement they may be employed with the Medical Service of the Egyptian Army, with the Sanitary Service of the Egyptian Government, or with the Medical Services of any foreign or Colonial Government. While in this position they are specially paid by the Government retaining them, but their service counts towards promotion and possibly pension in the R.A.M.C.

### Promotion.

In the matter of promotion in the R.A.M.C., an officer is eligible to pass to the rank of captain on the completion of three and a half years' service and to the rank of major on the completion of twelve years' total service, provided that in each case he has passed the necessary examination and is recommended for such promotion. The examination for captain's rank may be taken at any time after completing twelve months' service, and is confined in its subjects to practical tests in map reading and problems in connection with practical tactics, to military law, to organisation, administration, and equipment, and to military medical administration, the duties of executive medical officers, and the terms of the Geneva Convention. The major's examination may be taken after five years' service, and includes the subjects above mentioned, along with medicine, surgery, hygiene, bacteriology, and tropical diseases, together with one special subject, such as bacteriology, dermatology, advanced operative surgery, ophthalmology, otology, and several others. A knowledge, too, is required of R.A.M.C. drills and exercises. Above the rank of major promotion is not by any length of service, but by selection, and is dependent on vacancies. A major, however, before he can be promoted to lieutenant-colonel must pass a qualifying examination. This can be taken at any time after three years in the rank of major and is in the following subjects:—

1. Army medical organisation in peace and war.
2. Sanitation of towns, camps, transports, and all places likely to be occupied by troops in peace and war; epidemiology, and management of epidemics.
3. (*a*) Medical history of the more important campaigns, and the lessons to be learnt therefrom. (*b*) A knowledge of the Army Medical Service of the more important Powers. (*c*) The laws and customs of war so far as they relate to the sick and wounded.
4. A medical staff tour.

To assist candidates in the examination for promotion to the rank of major special facilities for study at the Royal Army Medical College are given and instruction in the special subjects.

In connection with this matter of promotion, there must be mentioned a very important concession, and that is that for distinguished service in the field, or for any other meritorious or distinguished service, including distinction in original investigation or research, a medical officer may be promoted to the next higher rank by brevet, which carries with it the pay and allowances of that rank.

### Retirement Regulations.

As regards retirement from the R.A.M.C., very definite rules are laid down. Thus, retirement may be compulsory from age, a surgeon-general having to go at sixty, a colonel at fifty-seven, and other officers at fifty-five, but it may also be voluntary, an officer being permitted to resign or retire at any time with the approval of the Army Council. The scale of retire-pay varies with rank and length of service. At present a director-general with three years' service in that appointment and thirty years' general service gets £1,125, a surgeon-general £730, a colonel with thirty years' service £547, a lieutenant-colonel £501, and a major after twenty years' service £375. Other officers with shorter periods of service and with lower rank receive gratuities on retiral. Thus, a major with six years' service in that rank gets £2,500, but with only three years' service £1,800, while an officer after five years' service in the rank of captain is entitled to £1,000. Of the officers thus voluntarily going into retirement, those with at least three, but not more than six, years' service may be allowed to join the reserve of officers for seven years, and they will receive while so situated a retaining fee of £25 a year.

Of the other conditions of service of an Army medical officer, it should be noted that he is entitled to sixty-one days of ordinary leave of absence on full pay during the year, and as regards sick leave, if granted on the recommendation of a Medical Board, he may draw full pay for a period not exceeding twelve months, which in special circumstances, such as loss of health due to tropical service or to active operations, may be extended, but it will not exceed eighteen months in all. Under certain conditions, too—wounds and injuries—pensions, as also gratuities, are granted to medical officers, and in the case of their dying, when either on the Active or Retired List, pensions may be granted to their widows and compassionate allowances to children.

A brief reference must be made to the military training. This is based on the principle that an Army Medical Service is maintained for two objects: first, the prevention of disease; and, secondly, the care and treatment of the sick and wounded. Thus no medical officer can carry out his duties efficiently unless he has a certain general knowledge of military science, especially as regards the administration of an Army in the field. Hence he must be competent to supervise all sanitary precautions, and be well acquainted with the system of collecting wounded. In addition to this a medical officer has his executive duties in connection with the men of his own corps that are under his command, and their needs and the maintenance of discipline amongst them call for his personal care. To qualify him for all these a system of individual and collective training has been laid down, the former comprising staff tours, war games, lectures, regimental exercises and indoor schemes, and essays, while in the latter are included regimental training, divisional and command training, and Army manœuvres.

[*Further details see page* 637.]

